# Investigating the Antiproliferative Activity of High Affinity DNA Aptamer on Cancer Cells

**DOI:** 10.1371/journal.pone.0050964

**Published:** 2013-01-16

**Authors:** Harleen Kaur, Jasmine J. Li, Boon-Huat Bay, Lin-Yue Lanry Yung

**Affiliations:** 1 Department of Chemical and Biomolecular Engineering, Faculty of Engineering, National University of Singapore, Singapore, Singapore; 2 Department of Anatomy, Yong Loo Lin School of Medicine, National University of Singapore, Singapore, Singapore; IDI, Istituto Dermopatico dell’Immacolata, Italy

## Abstract

Vascular endothelial growth factor (VEGF) is an angiogenic mitogen involved in promoting tumor angiogenesis inside the body. VEGF is a key protein required for progression of tumor from benign to malignant phenotype. In this study, we investigated the binding affinity of a previously selected 26-mer DNA aptamer sequence (SL_2_-B) against heparin binding domain (HBD) of VEGF_165_ protein. The SL_2_-B was first chemically modified by introduction of phosphorothioate linkages (PS-linkages). Subsequently, surface plasmon resonance (SPR) spectroscopy and circular dichroism (CD) were used to determine the binding affinity, specificity and to deduce the conformation of PS-modified SL_2_-B sequence. Finally, antiproliferative activity of the modified SL_2_-B sequence on Hep G2 cancer cells was investigated. Our results demonstrate a marked enhancement in the biostability of the SL_2_-B sequence after PS modification. The modified SL_2_-B sequence also exhibits enhanced antiproliferative activity against Hep G2 cancer cells in hypoxia conditions. In addition, modified SL_2_-B sequence inhibits the expression of Jagged-1 protein, which is one of the ligands to VEGF linked delta/jagged-notch signaling pathway.

## Introduction

Cancer is one of the leading causes of death worldwide and accounted for 7.6 million deaths in 2008 [1], [2]. In the United States alone, approximately 1 in 4 people die due to cancer [3]. Currently, monoclonal antibodies are one of the most advanced therapeutic agents for cancer treatment in the market. Several FDA approved monoclonal antibody drugs, such as bevacizumab (trade name: Avastin) against vascular endothelial growth factor (VEGF) in colorectal, lung, and kidney cancer treatment, trastuzumab (trade name: Herceptin) against HER2/neu receptor in breast cancer treatment, and cetuximab (trade name: Erbitux) against epidermal growth factor receptor (EGFR) in metastatic colorectal, head and neck cancers, have been developed and are used either as a single agent or in combination with other drugs and radiation for cancer therapy [4]–[12].

In 1990, an *in vitro* selection process called systematic evolution of ligands by exponential enrichment (SELEX) was developed to screen single stranded nucleic acid molecules from random pool of library against the target ligand [13], [14]. These classes of single stranded molecules are referred as “aptamers”. They possess high binding affinity and specificity that are comparable to monoclonal antibodies. In addition, the small size, non-immunogenicity and ease of modification compared to conventional monoclonal antibody makes aptamers attractive for therapeutic application [15]. Based on the promising results in preclinical studies, two cancer targeting aptamers, ACT-GRO-777 (or AS1411) - a G-rich DNA aptamer targeting nucleolin for treatment of acute myeloid leukemia (AML) and NOX-A12 L-RNA aptamer targeting CXCL12 for treatment of multiple myeloma and lymphoma are already in clinical trials [16], [17].

One chief problem that arises in the therapeutic application of aptamers is their instability under *in vitro* and *in vivo* conditions [18]. They are susceptible to enzymatic nuclease attack in the cellular and serum fluids. To circumvent this problem, several chemical modification strategies have been employed to enhance their resistance against nucleases and to prolong their circulation half-life in the biological fluids. Such chemical modifications include incorporation of phosphorothioate linkages (PS-linkages) or locked nucleic acids (LNAs), addition of functional groups such as amino (-NH_2_), fluoro (-F), *O*-methyl (-OCH_3_) in 2′-position of ribose sugar, and conjugation to high molecular mass polyethylene glycol (PEG) or cholesterol [19]–[25]. Studies have demonstrated that, compared to the unmodified version, the chemically modified aptamers exhibit not only longer lifetime in the biological milieu but sometimes also better binding affinity and specificity to their targets [21], [26].

VEGF is a crucial angiogenic mitogen overexpressed in the tumor cells and induces their migration, excessive proliferation, invasion and metabolism inside the body. VEGF is considered to be the hallmark protein for tumor angiogenesis and has been associated with neoplastic transformation of cells inside the body [27]. It is generally thought to be secreted by endothelial cells to stimulate their proliferation and migration. Previous reports, however, indicate that different carcinoma and malignant mesothelioma cell lines also secrete this protein [28]–[31]. VEGF_165_ is the pre-dominant isoform of VEGF-A protein, one of the members of VEGF family, and primarily binds to its two tyrosine-kinase receptors VEGFR-1/Flt-1 and VEGFR-2/KDR/Flk-1 with very high affinity and to specific co-receptor neuropilins [27]. The mitogenic signaling and cell proliferation in tumor cells is induced by expression of VEGFR-2 [32], [33]. In contrast, activation of VEGFR-1 results in cell invasion and cell migration but not cell proliferation [34]–[36].

In our previous study, a 26-mer DNA aptamer against heparin binding domain (HBD) of VEGF_165_ protein (referred to as SL_2_-B) was obtained using stem-loop truncation strategy [37]. Compared to the original untruncated aptamer, the SL_2_-B aptamer exhibited more than 200-fold increase in the binding affinity to VEGF_165_ protein. Herein, we modified the SL_2_-B aptamer by incorporating phosphorothioate (PS) linkages, tested its binding affinity, specificity, biostability, secondary structure and the potential feasibility of the PS-modified SL_2_-B aptamer as antagonist on the proliferation activity of cancer cells. We demonstrated that, compared to unmodified SL_2_-B aptamer, the PS-modified SL_2_-B aptamer is an improved sequence in terms of serum stability and antiproliferative activity without sacrificing the binding affinity and specificity for VEGF_165_ protein.

## Materials and Methods

### Materials

The HPLC purified oligonucleotide (both unmodified and PS-modified) was purchased from Sigma-Aldrich. The recombinant human carrier free VEGF_165_ (molecular weight of 38 kDa, pI = 8.25) and VEGF_121_ (molecular weight of 28 kDa, pI = 6.4) proteins were purchased from R & D systems. CM5 sensor chips were purchased from GE Healthcare for protein immobilization. 1-ethyl-3- [3-dimethylaminopropyl] hydrochloride (EDC), N-hydroxysuccinimide (NHS), and ethanolamine-HCl were purchased from Sigma-Aldrich. Sodium acetate (anhydrous) was purchased from Fluka. Tween-20 was purchased from USB Corporation. Acrylamide/Bis-acrylamide (30%) and triton X-100 were purchased from BIO-RAD. Sodium dodecyl sulfate (SDS), phosphate buffer saline (PBS), and sodium hydroxide (NaOH) were purchased from 1^st^ Base. Human hepatocellular carcinoma (Hep G2) cell line was a gift from Dr. Tong Yen Wah’s lab, which was purchased from ATCC. Human breast adenocarcinoma (MCF-7) cell line and human colorectal carcinoma cell line (HCT-116) were purchased from ATCC. The hypoxia chamber was purchased from Billups-Rothenberg. Dulbecco’s modified eagle’s media (DMEM) media, and fetal bovine serum (FBS) were purchased from Caisson laboratories. Trypsin-EDTA and 1% penicillin/streptomycin mixture were purchased from PAN biotech. Thiazolyl blue tetrazolium bromide (MTT, 97.5%) ammonium persulfate (APS), urea and N, N, N′, N′-methylene-bis-acrylamide (TEMED, 99%), nadeoxycholate and tris buffer were purchased from Sigma-Aldrich. Monoclonal anti-human Jagged-1 fluorescein antibody was purchased from R & D systems. Jagged-1 (28H8) rabbit monoclonal antibody was purchased from cell signaling. Purified mouse anti-calnexin antibody was purchased from BD transduction laboratories. The lysis and extraction buffer RIPA (Radio-Immunoprecipitation Assay) buffer for western blotting was prepared with the following reagents: RIPA Buffer (50 ml), 50 mM Tris (pH 7.8), 150 mM NaCl, 0.1% SDS (sodium dodecyl sulphate), 0.5% Nadeoxycholate, 1% Triton X-100, 1 mM phenylmethylsulfonyl fluoride (PMSF). One tablet of the protein inhibitor cocktail, complete mini tablet (Roche Applied Science, Switzerland) was dissolved in 10 ml of the buffer to complete the lysis buffer preparation. Polyvinyllidene difluoride (PVDF) membrane, wet pico chemiluminescence substrate and CL-exposure film were purchased from thermo scientific. The FITC annexin V apoptosis detection kit was purchased from BD Pharmingen, Germany. PMSF was purchased from CalBiochem.

### Surface Plasmon Resonance (SPR) Spectroscopy

**Figure 1:**
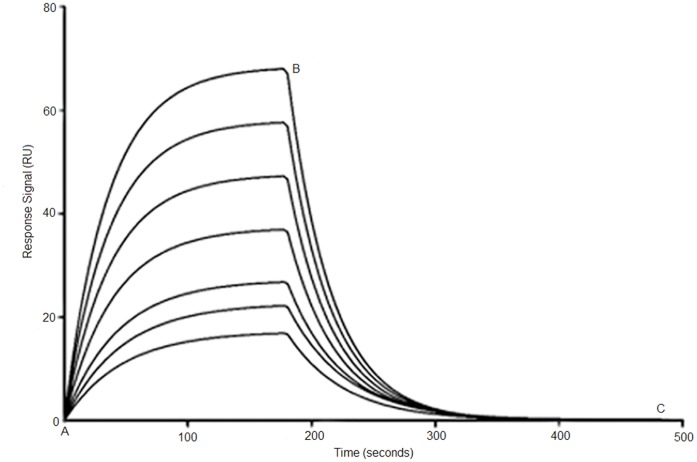
Typical SPR sensorgrams demonstrating interaction of aptamer with immobilized VEGF_165_ protein at different concentration (bottom to top, 0.2 to 100 **nM).** Point A to B corresponds to association phase and point B to C corresponds to the dissociation phase in all the sensorgrams. Shown here is PS-modified SL_2_-B aptamer (K_d_ = 0.56±0.44 nM).

The binding affinity and specificity of modified aptamer sequence was investigated using surface plasmon resonance (SPR) spectroscopy, where VEGF_165_ and VEGF_121_ acted as ligands and were directly immobilized on the sensor chip. Briefly, the carboxylic group on the sensor chip was activated by standard amine coupling procedure using freshly prepared EDC/NHS. VEGF_165_ or VEGF_121_ (25 µg/ml) in acetate buffer (pH 6.0) were then injected into the sensor chip at flow rate 8 µl/min to reach ∼200 RU immobilization level. The deactivation was done by ethanolamine-HCl to block unreacted carboxyl groups. The binding analysis was carried out with modified aptamers at different concentrations (0.2 to 100 nM) using a BIAcore 2000 instrument (GE Healthcare). The running condition was set at 30 µl/min flow rate, 25°C, 3 min association time and 5 min dissociation time. PBS and tween-20 solution mixture was used as the running buffer, and 50 mM NaOH as the regeneration buffer. All the buffers were filtered and degassed prior to each experiment. Blank surfaces were used for background subtraction. Upon injection of the aptamers, sensorgrams recording the association/dissociation behavior of the VEGF-aptamer complex were collected. By varying the aptamer concentration, a series of sensorgrams (Figure 1) were obtained and subsequently analyzed using the 1∶1 Langmuir model provided in the BIAevaluation software (version 4.1) to calculate the equilibrium dissociation constant K_d_. All SPR measurements were performed in triplicates.

### Stability of SL_2_-B Aptamer Against Nucleases in Serum Containing Medium

To test the stability of the unmodified and PS-modified SL_2_-B aptamer against nucleases, 10 µM aptamer was incubated for different time intervals in DMEM media supplemented with 10% FBS at 37°C. 25 µl of sample was taken out at different time point (0, 12, 24, 48, and 72 hours) and immediately stored at −80°C to minimize unnecessary degradation. Samples were then subjected to 12% denaturing polyacrylamide gel electrophoresis (PAGE). The band density was quantitatively measured using gel densitometry and analyzed using gene tools software from Syngene.

### Circular Dichroism (CD) Spectroscopy

To deduce the structure of PS-modified SL_2_-B aptamer, 10 µM of aptamer was dissolved in the PBS buffer for CD analysis. The CD spectrum was recorded in wavelength range of 200–320 nm at two different temperatures 25°C and 37°C and the data were the average of 10 scans. The CD spectrum analysis was performed using cuvette of 1-cm path length on a Jasco J-810 spectropolarimeter. The PBS buffer was used as blank for both the temperatures and the spectral data for SL_2_-B aptamer was blank corrected.

### Antiproliferative Activity Assay

Hep G2 and MCF-7 cells were seeded at a density of 2000 cells/ml and HCT-116 cells were seeded at a density of 3000 cells/ml in 96-well plate at day 0 in DMEM media supplemented with 10% FBS and penicillin/streptomycin mixture. SL_2_-B aptamer (unmodified/PS-modified) and scrambled aptamer were incubated with cells at different concentrations and incubated for 3 days in hypoxia conditions (5% CO_2_, 1% O_2_, and 94% N_2_) inside the hypoxia chamber. The cell medium was not changed for 3 days. No cell transfecting or permeabilizing agent was added. The antiproliferative effect of aptamer on the cells was determined by measuring cell viability using colorimetric MTT assay. The optical density reading was recorded using microplate reader (Tecan, infinite M200) at 570 nm with background subtraction at 620 nm. The experiment was performed in triplicates.

### Microscopy Imaging

The antiproliferative effect of PS-modified SL_2_-B aptamer on Hep G2 cells was assessed using optical microscopic imaging. Same conditions were maintained as for the antiproliferative activity assay and cells were imaged after 72 hours of aptamer treatment. Photomicrographs were taken on an Eclipse T5000 (Nikon, Japan) light microscope with Tame2u acquisition software.

### Apoptosis Assay

Annexin V apoptosis assay was performed to investigate the cell death mechanism in Hep G2 cells according to manufacturer’s protocol. Cells were harvested by trypsinization and washed twice with cold PBS (1X) and subsequently stained with FITC Annexin V and propidium iodide. Analysis was performed on the Beckman-Couter CyAn™ ADP flow cytometer by counting 15000 events.

### Flow Cytometry Analysis

Flow cytometry was used to study the effect of PS-modified SL_2_-B aptamer on Jagged-1 protein expression in Hep G2 cells. Hep G2 cells were seeded at a density of 80,000 cells/ml in 6-well plate at day 0 in DMEM media supplemented with 10% FBS and penicillin/streptomycin mixture. Following day after seeding, the cells were treated with modified SL_2_-B aptamer and scrambled aptamer sequence at 15 µM aptamer concentration. Same hypoxia conditions were maintained as for the antiproliferative activity assay. After 3 days of aptamer treatment, the cells were trypsinized, incubated with anti-human Jagged-1 fluorescein antibody for 1 hour, re-suspended in PBS buffer and analyzed immediately using a Beckman-Couter CyAn ADP flow cytometer by analyzing 15,000 events and relative fluorescence was determined using SUMMIT V 4.3.02 software.

### Western Blot Analysis

The sequence specific effect of PS-modified SL_2_-B aptamer on Jagged-1 protein expression in Hep G2 cells was analyzed using western blotting. Same experimental conditions were maintained as for the flow cytometry. After 3 days of aptamer treatment, the cell medium was removed and the cells were washed once in cold 1×PBS. 500 µl of the complete lysis buffer was added to each 6-well and the cells were scrapped with a cell scrapper and collected into microcentrifuge tubes. The extracted proteins were resolved on an SDS-PAGE gel and transferred onto a PVDF membrane via wet transfer. Membranes were blocked in 5% non-fat milk and washed in tris-buffered saline with 1% tween. Subsequently, membranes were incubated with primary antibody (Jagged-1 rabbit monoclonal and purified mouse anti-calnexin antibody) and then with corresponding secondary antibody (goat anti-rabbit and anti-mouse IgG secondary antibody conjugated to horseradish peroxidase (HRP)) with 3 washing steps in between. The protein bands were developed with west pico chemiluminescence substrate and visualized on XPress CL blue ray film. Optical densities of bands were measured on a GS800 densitometer and band intensities were analyzed with Quantity One image analysis software (Biorad, USA).

### Statistical Analysis

Data are presented as mean ± SD. A p-value <0.05 was considered statistically significant using student’s t-test.

## Results and Discussion

### Binding Analysis of PS-modified SL_2_-B Aptamer and VEGF Complex by Surface Plasmon Resonance (SPR)

As reported previously in our study, the unmodified SL_2_-B aptamer displayed a K_d_ = 0.5 nM to heparin binding domain (HBD) of VEGF_165_ protein determined via SPR technique (Table 1) [37]. The unmodified aptamer, however, exhibited low structural stability in the cellular conditions. This is due to the presence of exonucleases and endonucleases in biological fluids which degrade the aptamers by hydrolyzing the phosphate ester bond in the backbone [19]. To alleviate this problem, in this study, the SL_2_-B aptamer was chemically modified with phosphorothioate (PS) linkages at 5′ and 3′- terminus (Table 1) to protect the SL_2_-B aptamer from exonucleolytic digestion. The PS-modification involves the substitution of unbridged phosphoryl oxygen in phosphodiester linkage by sulfur atom. Since the excess incorporation of PS-linkages leads to non-specific binding and can perturb the aptamer conformation and its interaction with the target, the modification was introduced only at aptamer termini [38].

**Table 1:** Unmodified and PS-modified SL_2_-B aptamer sequences along with their equilibrium dissociation constant (K_d_) values determined using surface plasmon resonance (SPR) spectroscopy.

Sequences of original and PS-modified aptamer (5′–3′)	K_d_
**Unmodified SL_2_-B aptamer** CAATTGGGCCCGTCCGTATGGTGGGT	0.50±0.32 nM
**PS-modified SL_2_-B aptamer** C*AATTGGGCCCGTCCGTATGGTGGG*T	0.56±0.44 nM

“*” indicates the position of phosphorothioate (PS) modification.

The K_d_ value for PS-modified SL_2_-B aptamer was determined using SPR technique at different aptamer concentrations (Figure 1 and Table 1). The K_d_ value for the PS-modified SL_2_-B was found to be 0.56 nM, which is similar to the K_d_ for unmodified SL_2_-B. Introducing PS-modification does not appear to affect the binding affinity of the SL_2_-B aptamer. Moreover, the affinity of PS-modified SL_2_-B is similar to the FDA approved humanized anti-VEGF monoclonal antibody “bevacizumab” (K_d_ ∼ 0.5 nM) used for cancer treatment [4].

### Specificity of PS-modified SL_2_-B Aptamer Sequence

VEGF_165_ as well as other VEGF isoforms, such as VEGF_189_ and VEGF_206_, are generated from splicing of a single VEGF gene that shares a carboxyl-terminal heparin-binding domain (HBD) of 50-residues and binds to heparin with different binding affinities [27], [39], [40]. HBD is responsible for enhancing the interaction of VEGF with its receptors (VEGFR-1/Flt-1 and VEGFR-2/KDR/Flk-1) and the specific co-receptor neuropilins to trigger the angiogenic response in malignant cells [41].

**Figure 2:**
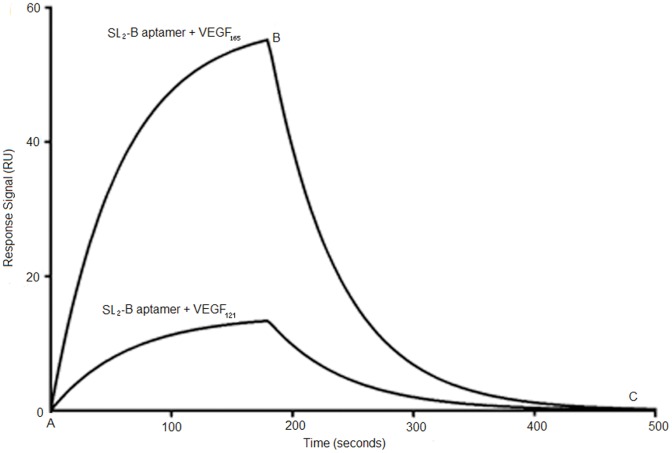
SPR sensorgrams demonstrating interaction of PS-modified SL_2_-B aptamer with immobilized VEGF_165_ and VEGF_121_ protein at same concentration. Point A to B corresponds to association phase and point B to C corresponds to the dissociation phase in both the sensorgrams. Shown here is PS-modified SL_2_-B aptamer binding with VEGF_165_ protein (K_d_ = 0.56±0.44 nM) and VEGF_121_ protein (K_d_ = 17±1.24 µM) at 80 nM aptamer concentration.

VEGF_121_, however, does not share the HBD as other VEGF isoforms and can be used as a control for HBD binding specificity study. The SPR sensorgram in Figure 2 shows that compared to VEGF_165_ protein at same aptamer concentration (80 nM), the response signal of PS-modified SL_2_-B binding to VEGF_121_ protein was weak and displayed a high K_d_ value of 17 µM. This indicates that PS modification does not reduce the binding specificity of SL_2_-B aptamer towards HBD significantly (K_d_ = 17 µM for PS-modified SL_2_-B towards VEGF_121_, K_d_ = 10 µM for unmodified SL_2_-B towards VEGF_121_). Compared to the “bevacizumab” monoclonal antibody that binds to all isoforms of VEGF, the PS-modified SL_2_-B is specific to HBD of VEGF_165_ protein [4]. Since VEGF-A is involved in normal physiological processes, such as formation of new blood vessels and wound healing process, the complete inhibition of VEGF protein can affect the maintenance of the normal vascular system inside the body [42], [43]. Therefore, inhibition of specific VEGF protein (for example, VEGF_165_ in this case) may be a better therapeutic approach.

### Stability of SL_2_-B Aptamer Against Nucleases in Serum Containing Medium

**Figure 3:**
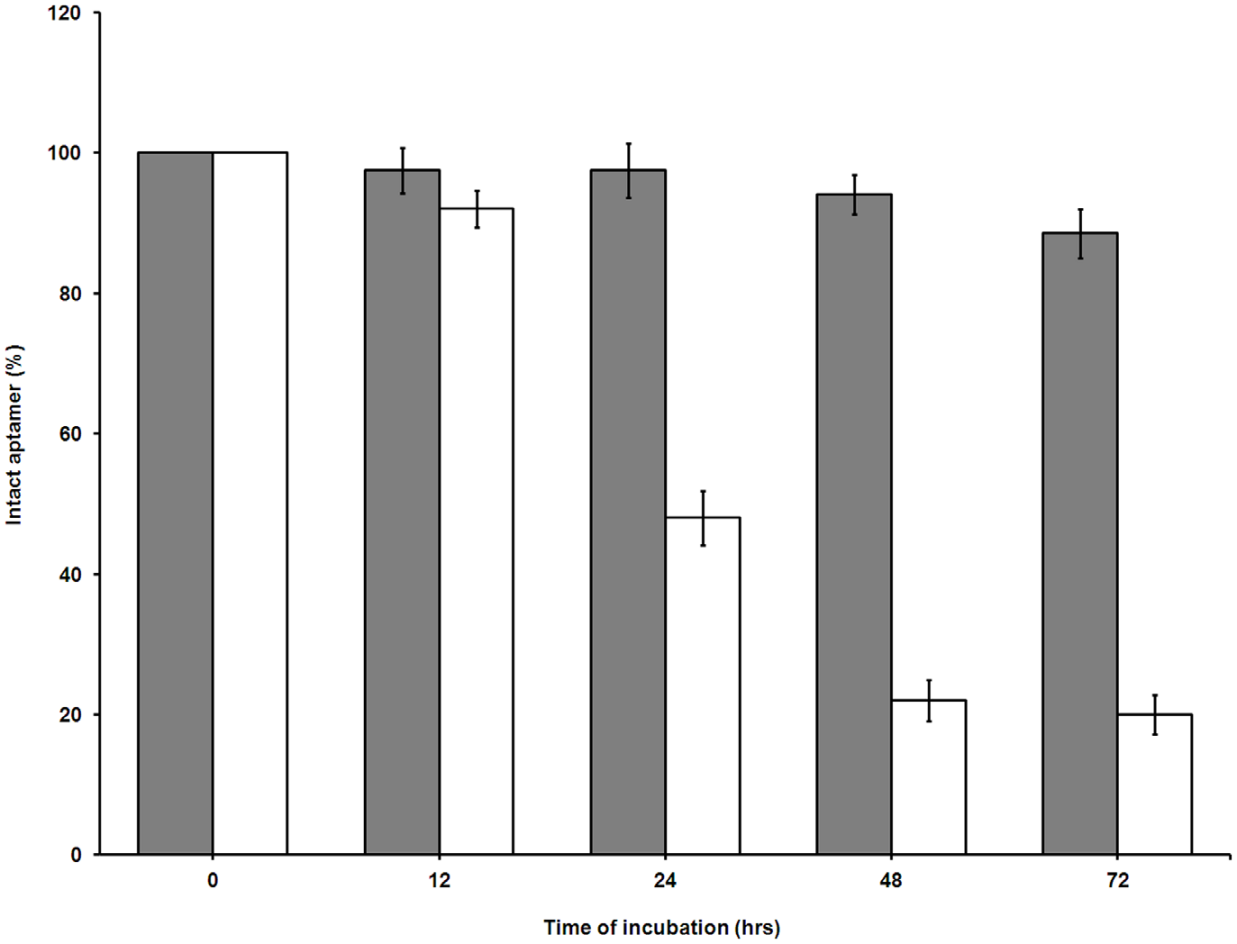
Nuclease-resistance stability of unmodified and modified SL_2_-B aptamer sequence in 10% FBS. Aptamers were incubated with 10% FBS dissolved in DMEM media at 37°C for different time points and percentage of intact aptamer was determined by measuring the band density after running denaturing PAGE. Filled columns are PS-modified SL_2_-B, while open columns are unmodified SL_2_-B.

To test the biostability of the unmodified and PS-modified SL_2_-B aptamer against nucleases present in the biological fluids, both aptamers were incubated with 10% FBS for different time periods. Based on the results, the unmodified SL_2_-B degraded by 50% within 24 hours of incubation in serum (Figure 3). On the other hand, the PS-modified SL_2_-B displayed good stability, with more than 90% aptamer intact after 72 hours of incubation in the serum. The data demonstrates the importance of PS-linkages in the SL_2_-B sequence termini, which protects the aptamer sequence from exonuclease attack.

### Structural Analysis by Circular Dichroism (CD) Spectroscopy

**Figure 4:**
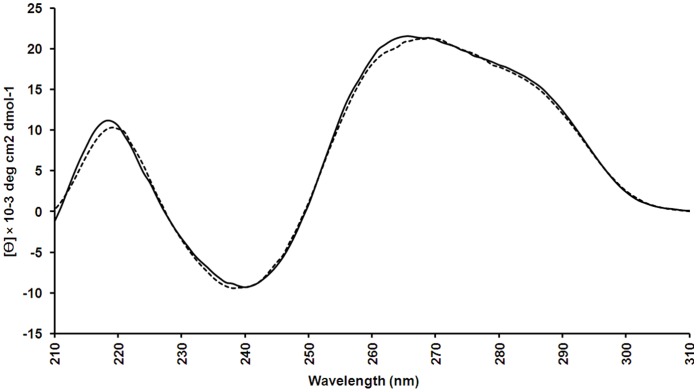
CD spectra of 10 µ**M PS-modified SL_2_-B aptamer in phosphate buffer saline (PBS) buffer, pH-7.2. Spectra were measured at 25°C (solid line) and 37°C (dotted line).**

Structural studies have shown the impact of the conformation on the binding affinity and specificity of the aptamer for its target [44]. If the conformation changes with temperature, then the binding affinity results obtained from SPR spectroscopy (conducted at 25°C) may not be representative in *in vitro* assays (conducted at 37°C). Thus, the secondary conformation of the PS-modified SL_2_-B aptamer was investigated. Positive maxima peaks were observed at 260 nm and 220 nm as well as a negative minima peak at 240 nm and additional small shoulder peak at 290 nm (Figure 4). Based on the previous reports, such spectra reflect a typical hairpin stem-loop conformation [45]. Since no change in the spectra was observed between 25°C and 37°C, this confirms the preservation of the secondary conformation at the SPR conditions (25°C) where the K_d_ of the aptamer was determined and at physiological conditions (37°C). However, the CD spectroscopy does not provide the complete and validated information on the structure. Advanced techniques such as nuclear magnetic resonance (NMR) and X-ray crystallography are required for further in-depth structural analysis.

### Antiproliferative Activity Assay

**Figure 5:**
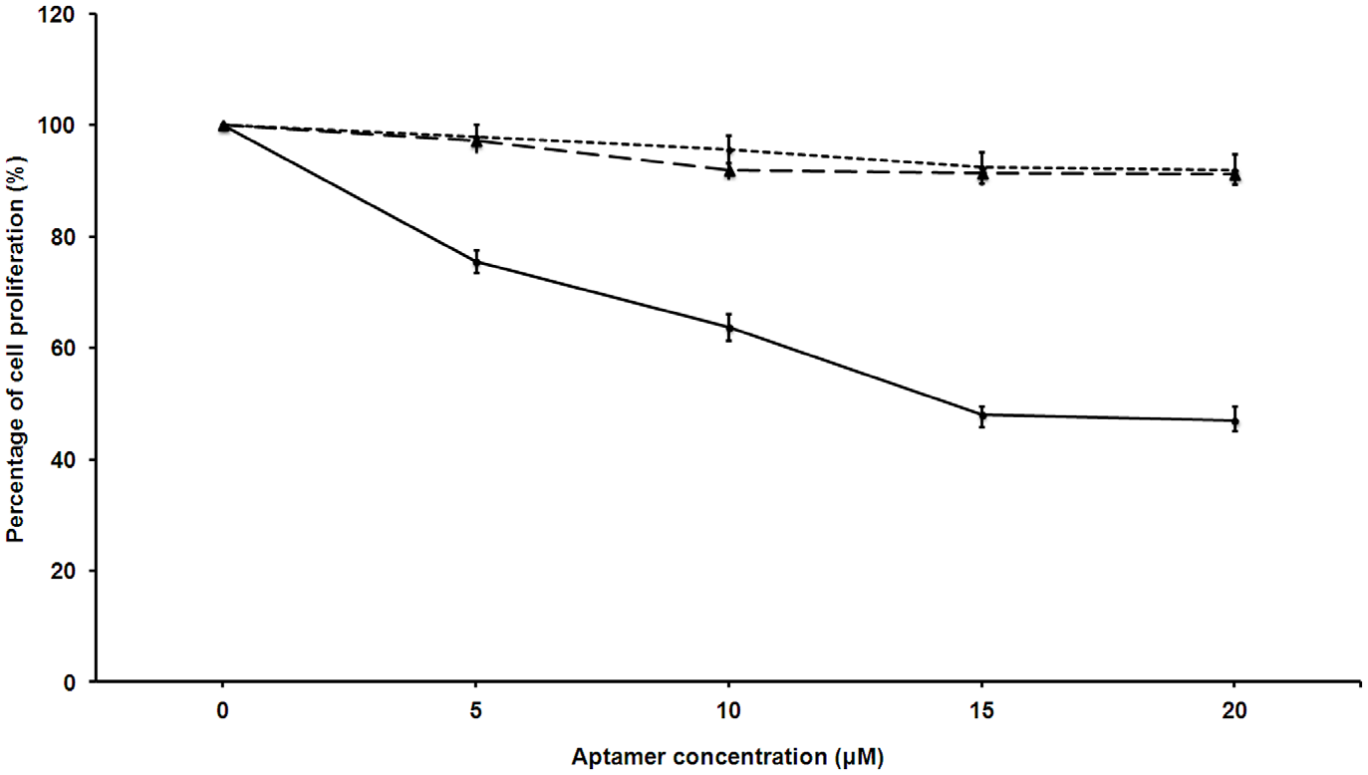
Relative % proliferation of Hep G2 cells (compared to control) after treating with unmodified and PS-modified SL_2_-B aptamers at different concentrations in hypoxia conditions. The sequence specificity was determined using scrambled sequence for PS-modified SL_2_-B for each data point at same concentration to the modified SL_2_-B. Solid line is PS-modified SL_2_-B, dashed line is unmodified SL_2_-B, and dotted line is scrambled sequence.

The antiproliferative property of SL_2_-B aptamer was studied using Hep G2 cancer cells in hypoxia conditions. Previous studies have demonstrated that the expression of VEGF protein is potentiated in Hep G2 cells under hypoxia conditions [46]. Since no significant effect on cell proliferation was observed at 24 and 48 hours, both the unmodified and PS-modified SL_2_-B aptamers were tested for 72 hours duration. As shown in Figure 5, lower cell proliferation was observed at 15 µM modified SL_2_-B concentration after 72 hours of aptamer treatment (52±2.1%). However, no decrease in the cell proliferation was observed on further increasing aptamer concentration to 20 µM. A possible explanation for decrease in the cell proliferation could be that either the excess binding of modified SL_2_-B sequence to VEGF_165_ protein ultimately prevents the interaction of the protein to the VEGFR-2 (or KDR/Flk-1) receptor, which affects the cellular proliferation. Or aptamer after binding with VEGF protein binds with VEGFR-2, undergoes cellular internalization and interferes with the downstream VEGF linked intracellular signaling pathways. The result also indicates that VEGF protein may be involved in the proliferation of investigated Hep G2 cancer cells under hypoxia conditions. On the contrary, the unmodified SL_2_-B aptamer sequence did not exhibit significant inhibitory activity on the cellular proliferation. This could be due to the degradation of the unmodified sequence by nuclease enzymes in the media before pronouncing its effect on the cancer cells.

**Figure 6:**
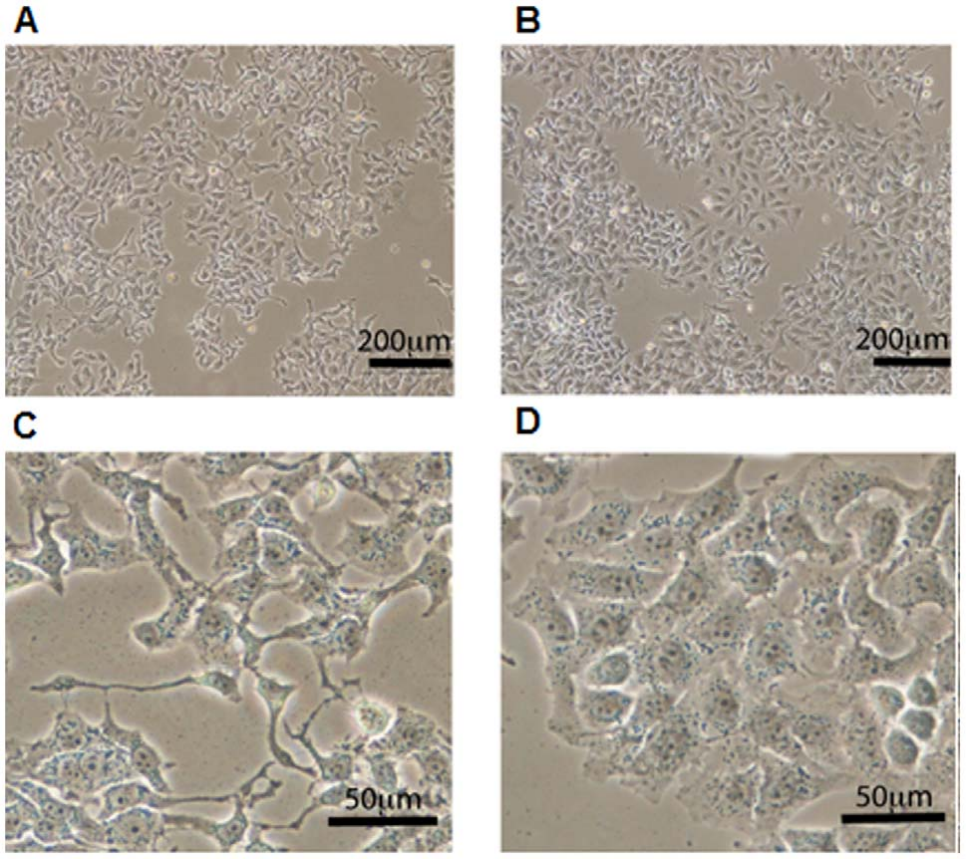
Effect of PS-modified SL_2_–B aptamer sequence compared to the scrambled sequence on Hep G2 cells. Low magnification view of (A) modified sequence treatment, (B) scrambled sequence treatment on Hep G2 cells after 72 hours under hypoxia condition. Scale bar = 200 µm. Close up views of (C) modified sequence treatment, (D) scrambled sequence treatment on Hep G2 cells after 72 hours under hypoxia condition. Cellular morphology differs upon the different treatments; modified sequence treatment produces cells which are thinner with more cellular projections while the scrambled sequence treatment shows cells which appear closer to the untreated Hep G2 cells. Scale bar = 50 µm.

To demonstrate that the antiproliferative effect of PS-modified SL_2_-B aptamer is sequence specific, a scrambled sequence was added to the Hep G2 cells at the same concentration as PS-modified SL_2_-B (Figure 5). The results showed minimal decrease on the cell proliferation with the scrambled sequence, confirming that the inhibitory effect on VEGF_165_ protein activity by PS-modified SL_2_-B was sequence specific in Hep G2 cells. The sequence specific inhibition was also confirmed by the cell count and morphological differences presented in photomicrographs (Figure 6). As shown in Figure 6A and 6B, the cells treated with modified sequence have noticeably fewer cells as compared with the scrambled sequence where there appears to be more cells per view and packed closely to one another. Furthermore, under the same magnification, the morphology of the cells treated with the modified sequence appears longer and thinner with many side projections as compared with the scrambled sequence, which are more angular and more defined in shape (Figure 6C and 6D). These findings indicate the potential of the PS-modified SL_2_-B aptamer sequence in inhibiting the Hep G2 cancer cells proliferation strongly and specifically.

**Figure 7:**
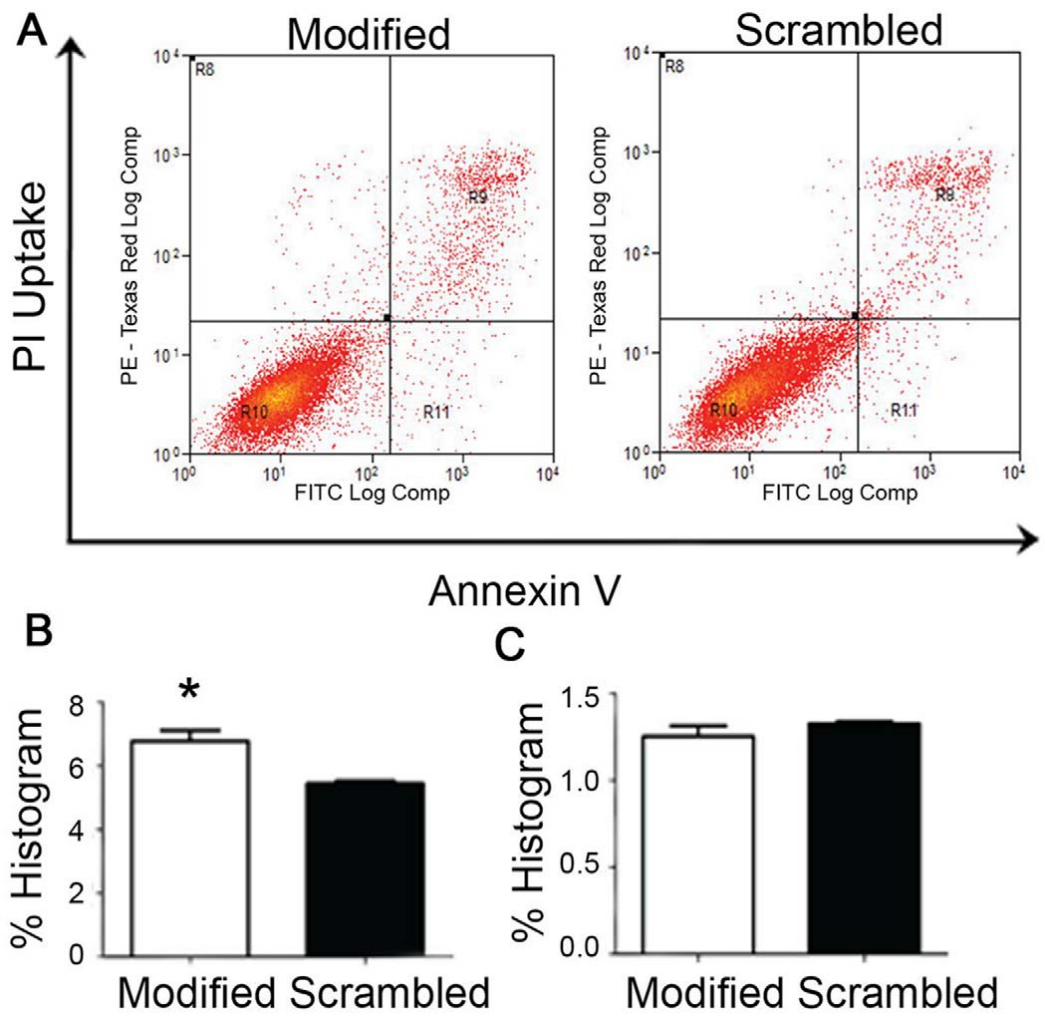
Annexin V assay of Hep G2 cells treated with modified sequence and scrambled sequence. (A) The scatterplot depicting the distribution of cells with annexin V staining along the x-axis and those stained with propidium iodide (PI) along the y-axis. Region R10 denotes the viable population (double negative for annexin V and PI), R9 the non-viable cells (double positive for annexin V and PI), R11 shows the annexin V positive (PI negative) population while R8 are the damaged cells (PI positive but annexin-V negative). (B) % Histogram of the R9 quadrant data. The analysis of the triplicate samples for showed a significantly higher amount of dead cells (p-value <0.05) in the modified sequence treatment compared to the scrambled sequence control. (C) % Histogram of R11 quadrant data. The results show no significant difference for early apoptosis. Error bars = SEM.

To determine the cell death mechanism in Hep G2 cells, annexin V apoptosis assay was performed and analyzed using flow cytometry. In Figure 7A, the R9 and R11 quadrant cells in flow cytometry scatterplot were counted and expressed as percentage of cells in late and early apoptosis phase respectively. Early apoptotic cells include cell population that is annexin V positive only (R11), and late apoptotic cells include cell population that is both annexin V and PI positive (R9). The apoptosis assay showed increased percentage of cell death with modified sequence compared with the scrambled sequence treatment in late apoptosis phase (Figure 7B, p-value <0.05). However, the percentage of cells undergoing late apoptosis was not very high and no significant difference in cell count was observed between modified and scrambled sequence in early apoptosis phase (Figure 7C). This result indicates that besides apoptosis, other non-apoptotic cell death mechanism such as senescence may be involved in induction of cell death in the Hep G2 cells.

To confirm the antiproliferative ability of the PS-modified SL_2_-B aptamer, we further investigated the effect with MCF-7 cells and HCT-116 cells since existing literature has shown that they also overexpress VEGF protein in hypoxia conditions [47], [48]. A 15 µM modified SL_2_-B concentration was used in this study but our results showed that both MCF-7 and HCT-116 cancer cells displayed only 23±3.2% and 9±1.8% decrease in cell proliferation was observed respectively. Based on these cell proliferation results, the effect of PS-modified SL_2_-B sequence on cell proliferation is believed to be cell type specific. Since antiproliferative effect on MCF-7 and HCT-116 cancer cells were not very substantial, they were not used for further studies below. Additional antiproliferative studies on various cancer cell types should be conducted to uncover the potential therapeutic targets and to identify the factors responsible for cell specific antiproliferative activity of this aptamer.

### Flow Cytometry and Western Blot Analysis of Jagged-1 Protein Expression

**Figure 8:**
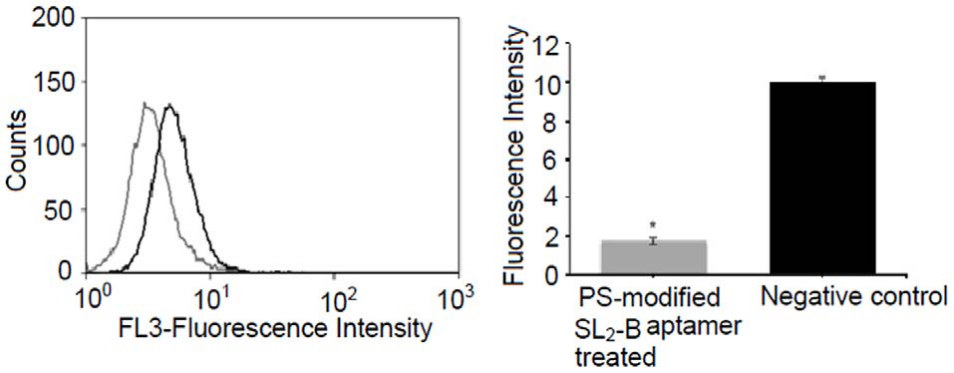
Flow cytometry histogram of Jagged-1 protein expression in Hep G2 cells using anti-human Jagged-1 antibody and quantitative analysis of flow cytometry result. Each histogram curve represents the expression of Jagged-1 obtained with (gray line) and without (black line, negative control) treatment with PS-modified SL_2_-B aptamer at 15 µM concentration. *Significant difference from the negative control sample at p-value <0.05.

Notch signaling is an evolutionary conserved signaling pathway affecting many cellular processes such as cell-fate determination, differentiation, proliferation, and survival. Five Notch ligands (Jagged-1, Jagged-2, Delta-1, Delta-3, and Delta-4) and four Notch receptors have been well established in mammals [49], [50]. Evidence indicates the biochemical linkage between VEGF and delta/jagged-notch pathways activation, and together both are involved in promoting tumor progression [51], [52]. In this linkage, VEGF pathway is essential for the initiation of tumor angiogenesis and acts as the upstream activating stimulus, whereas notch signaling which acts on downstream of the VEGF pathway, helps to respond to activating stimulus and shape the activation by making cell fate decisions [49]. Due to the crosstalk between VEGF and notch signaling pathways, the effect of PS-modified SL_2_-B aptamer was tested on Jagged-1, which is one of the notch ligands. Jagged-1 is overexpressed in various malignant tumors and has been associated with cancer recurrence [53]–[55]. Here, we examined the effect of PS-modified SL_2_-B aptamer on the expression of Jagged-1 protein in Hep G2 cells via flow cytometry technique. Compared to the untreated sample (only cells), modified SL_2_-B treatment exhibited decrease in the fluorescent signal (Figure 8). This shift in the peak indicates the downregulation of the Jagged-1 expression due to the addition of PS-modified SL_2_-B aptamer in Hep G2 cells (p-value <0.05).

**Figure 9:**
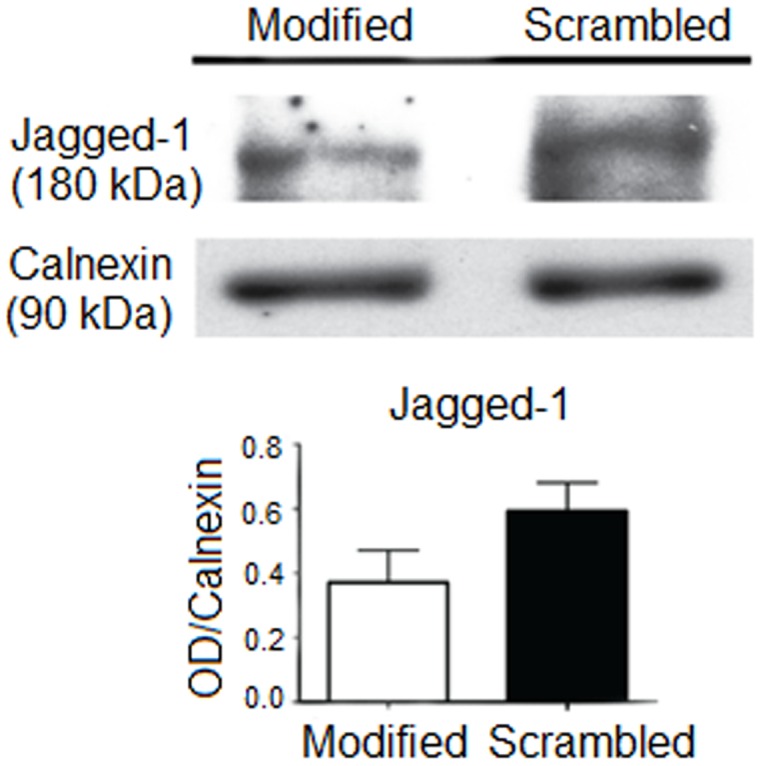
Western blot of whole cell lysates from Hep G2 cells treated with the PS-modified SL_2_ aptamer and scrambled sequence (control). The expression of Jagged-1 protein in Hep G2 cells was assessed. Calnexin protein was used as a loading control. Error bar = SEM.

Besides flow cytometry, the effect of PS-modified SL_2_ aptamer on Jagged-1 protein expression in Hep G2 cells was analyzed using western blotting. The scrambled sequence of the modified aptamer was used as control. The modified aptamer appears to induce a lower expression of the Jagged-1 protein in Hep G2 cells as compared to the scrambled sequence (Figure 9). This confirms the sequence specific inhibition of the aptamer on Jagged-1 protein expression in Hep G2 cells. Based on both flow cytometry and western blotting results, it can be concluded that the binding of PS-modified SL_2_-B aptamer to VEGF protein exhibits its antiproliferative activity in Hep G2 cells not only by inhibiting VEGF pathway but also the interconnected delta/jagged-notch signaling pathway in Hep G2 cells. Further studies are warranted to determine the effect of the modified aptamer on different notch ligands and other VEGF linked signaling pathways.

### Conclusions

To summarize, this work attempted to study the antiproliferative potential of SL_2_-B aptamer in cancer cells. From the data, we conclude that post-modification, the PS-modified SL_2_-B aptamer retained its binding affinity and specificity for the heparin-binding domain (HBD) of VEGF_165_ protein. Furthermore, compared to the unmodified aptamer, the modified SL_2_-B demonstrated good biostability and exhibited its sequence specific antiproliferative activity on Hep G2 cancer cells in hypoxia conditions. Thus, based on the results of this work, it appears that chemical modification can be a useful approach in prolonging the half-life of the SL_2_-B aptamer in the *in vitro* conditions. This newly obtained SL_2_-B aptamer sequence can potentially be useful in oligomer-based cancer therapeutic applications, though further preclinical studies are required for better understanding of the SL_2_-B aptamer sequence and to evaluate its potential therapeutic value for cancer treatment.
